# Validation and standardization of the Childhood Trauma Screener (CTS) in the general population

**DOI:** 10.1186/s13034-022-00506-6

**Published:** 2022-09-01

**Authors:** Andreas Witt, Yusuf Öz, Cedric Sachser, Elmar Brähler, Heide Glaesmer, Jörg M. Fegert

**Affiliations:** 1 Department of Child and Adolescent Psychiatry/Psychotherapy, Ulm University, Steinhövelstr.1, 89075 Ulm, Germany; 2 Integrated Research and Treatment Center Adiposity Diseases, Department of Psychosomatic Medicine and Psychotherapy, University of Leipzig Medical Center, Leipzig, Germany; 3 Department of Psychosomatic Medicine, University Medical Center of Johannes Gutenberg University Mainz, Mainz, Germany; 4 Department of Medical Psychology and Medical Sociology, University of Leipzig, Leipzig, Germany

**Keywords:** Childhood Trauma Screener (CTS), Validation, Standardization, Screening, Normative data, Child maltreatment

## Abstract

**Background:**

A valid, quick and widely applicable retrospective screening tool for child maltreatment is of great importance to better adapt interventions and treatments. The Childhood Trauma Screener (CTS)*, derived from the Childhood Trauma Questionnaire (CTQ),* is one such instrument that aims to increase the likelihood of detecting mental and physical disorders that have manifested in adulthood as a result of traumatic experiences and maltreatment in childhood and adolescence. The present study aimed to investigate the psychometric properties of the CTS and generate normative data.

**Methods:**

Data from two representative surveys were combined. Both surveys used identical methods. The CTS, consisting of five items, other self-report instruments, and demographic characteristics were used. Construct validity was examined using confirmatory factor analysis (CFA). A subsample was used to examine convergent validity with the Adverse Childhood Experiences Questionnaire (ACE). Normative data are reported for age groups and gender.

**Results:**

A total of 5039 study participants provided valid responses to the 5-items questionnaire (54.3% female, response rate = 78.9%). CFA showed good fit indices for a 2-factor solution. Convergent validity was generally supported by moderate intercorrelations with the ACE.

**Conclusions:**

The results confirm the solid psychometric properties of the CTS as an easy-to-use, ultra-short retrospective measure of child maltreatment. The data can be used to compare sample or individual results with reference data provided.

**Supplementary Information:**

The online version contains supplementary material available at 10.1186/s13034-022-00506-6.

## Background

Child maltreatment is common and a risk factor for various developmental outcomes. Studies show that the consequences of child maltreatment are diverse [5] and may persist into adulthood [7, 10]. Besides mental disorders, such as posttraumatic stress disorder, depression and behavioral problems, consequences may include chronic somatic diseases [9] and may cause a high economic burden [8, 14]. Additionally, a range of meta-analyses have documented the impact of child maltreatment on neurobiological alterations [15, 23, 24, 29, 30]. Therefore, the reliable and economic assessment of child maltreatment is essential to various settings and research questions. To better put the results of specific populations or individuals into context, normative data is necessary.

A range of measures is available for the retrospective assessment of child maltreatment. One of the most widely used is the short version of the Childhood Trauma Questionnaire (CTQ) [4]. The short form of the CTQ consists of 28 items, measuring five subtypes of maltreatment: emotional abuse, physical abuse, sexual abuse, emotional neglect and physical neglect. Participants are required to rate abuse and neglect events on a 5-point Likert Scale (1- never true to 5-very often true). The CTQ is applied in clinical and non-clinical settings and is generally considered a reliable and valid instrument for the retrospective assessment of maltreatment [12].

The CTQ has been further reduced to a five-item screening version, the childhood Trauma Screener (CTS) [13], for more practical, cost-effective and accessible and therefore less error-prone use. Each of the five items assesses one subtype of maltreatment. The final five items of the CTS were derived based on their psychometric properties in a study, including a representative sample. One item of each of the five CTQ subscales was chosen based on values of discriminatory power, explained variance and feasibility to best represent the subscale [13]. The correlations between the items and the respective subscale ranged between r = 0.55 and r = 0.87. The internal consistency was acceptable (α = 0.76). The authors concluded that the CTS represents a reliable and very economic and straightforward screening tool for the retrospective assessment of child maltreatment [13]. Such brief screening instruments can economically assess covariates in studies or in stepped diagnostical approaches [12]. Therefore Glaesmer and colleagues provided clinical cut-offs based on a representative general population study in Germany [12]. However, the CTS has only been evaluated in the context of the CTQ. The five items of the CTS have never been examined independently. The five items of the CTS might show different psychometric properties when presented isolated compared to when presented in the context of the CTQ. Therefore, the present study aims to evaluate and validate the CTS based on two representative samples and provide normative data.

## Methods

### Study design and participants

Two representative population-based surveys were conducted in 2013 and 2018 in a three-stage approach by a research institute (USUMA) using identical procedures. The surveys were conducted in collaboration of different research groups focusing on health and wellbeing in the general population. As the two studies were conducted with identical procedures, data of these were combined to achieve a maximum of statistical power with a large sample size. In the first step, systematic area sampling was conducted based on the municipal classification of the Federal Republic of Germany (ADM F2F Sampling Frame). In doing so around 53,000 areas all over Germany were delimited electronically, these contained an average of around 700 private households in each area. These areas were then first layered regionally according to districts into a total of around 1500 regional layers and then divided into 128 disjunct “networks”. Each network served as a sampling frame, containing 258 single sample points proportionate to the distribution of private households in Germany. In the second stage, private households were systematically selected with a random route procedure [17] at each sample point. Households of every third residence in a randomly selected street were invited to participate in the study. In the third stage, in multi-person households, a kish-selection grid was used to ensure random participation. This means that to determine the target person, all members of the household who are 14 years and older are first entered into a scheme on the address list: all men who live in the household and are at least 14 years old are entered in descending order according to their age in boxes (e.g. 1 to 4) and all women are also entered in descending order according to their age in boxes (e.g. 5 to 8). The person whose number appears first in the sequence of random numbers is then to be interviewed, whereby the respective order of the random numbers varies in the data collection protocols. In this way, the target person is selected completely independent of the interviewer and the contact person [20].

Participants had to be at least 14 years of age and have sufficient German language skills. The potential participants were informed that the study was about health and well-being. Informed consent was obtained from those who indicated willingness to take part. The overall response rate was 78.9%.

Anonymity for saving the data and analyzing the data was guaranteed. After collecting sociodemographic data through a face-to-face interview, the researcher handed the questionnaires to the participant along with an envelope to seal afterwards, and then left the room but stayed nearby in case help was needed. The completed questionnaires were linked to the respondents' demographic data, but did not contain their name, address or other identifying information. Both surveys were conducted in accordance with the Declaration of Helsinki. They fulfilled the ethical guidelines of the International Code of Marketing and Social Research Practice of the International Chamber of Commerce and the European Society of Opinion and Marketing Research. Both surveys obtained ethics approval from the ethics committee of the Medical Faculty of the University of Leipzig before being carried out.

### Measures

Survey participants completed the Childhood Trauma Screener (CTS) [13]. The CTS consists of five items, which are: When I was growing up…

1. I felt loved (R) (emotional neglect)
2. There was someone to take me to the doctor when I needed it (R) (physical neglect)
3. People in my family hit me so hard, it left me with bruises or marks (physical abuse)
4. I felt that somebody in my family hated me (emotional abuse)
5. Somebody molested me (sexual abuse)

Respondents rate the items on a five-point Likert-Scale (1- never true to 5-very often true). The items can be used independently and a total score of all five items can be calculated ranging from five to 25. Additionally, we investigated a subscale for neglect, consisting of the two items for emotional and physical neglect with scores ranging from two to ten and the abuse subscale including the three items for emotional, physical and sexual abuse, with scores ranging from three to 15. This subdivision follows the Centers for Disease Control (CDC) definition of child maltreatment in the US [22].

Sociodemographic characteristics such as age, gender, education, marital status, employment status, net household income, nationality, place of residence, and religious affiliation were collected in a face-to-face interview. To assess convergent validity, part of the sample also completed the German version of the Adverse Childhood Experiences (ACE) questionnaire (ACE-D, [31]. This questionnaire consists of 10 items assessing adverse childhood experiences, including emotional abuse, physical abuse, sexual abuse, emotional neglect, physical neglect, parental separation, domestic violence, substance abuse, and incarceration of a household member. Items are scored dichotomously whether or not participants experienced these adverse childhood experiences in childhood.

### Statistical analyses

Item characteristics of the CTS items, including item means and item-intercorrelations, were examined. For reliability, the internal consistency (Cronbach's α) of the CTS total scale and the abuse and neglect subscales was assessed. For factorial validity, the factor structure of the CTS was investigated using confirmatory factor analysis (CFA) [18]. To assess dimensionality, CFAs were used to examine a two-dimensional structure of the CTS representing two subscales and a one-dimensional structure representing the total CTS score. Factorial invariance was tested between two subsamples divided by gender. We used five criteria to assess how well the model fits the data [18]. Three of these criteria indicate the absolute model fit: the root mean square error of approximation (RMSEA), the 90% confidence interval for RMSEA, and Standardized Root Mean Square Residual (SRMR). The other two criteria represent measures of relative model fit: the Comparative Fit Index (CFI) and the Tucker Lewis Index (TLI). RMSEA < 0.05 represents a “close fit”, RMSEA between 0.05 and 0.08 represents a “reasonably close fit”, and RMSEA > 0.10 represents an “unacceptable model” [18]. SRMR of 0 represents a perfect fit, SRMR < 0.05 represents a good fit, and an SRMS between 0.05 and 0.10 represents an adequate fit [18]. CFI and TLI indicate how well a given model fits the data relative to a “null” model, which assumes that sampling error alone explains the covariation among the observed measures. Hu and Bentler [18] have suggested that measurement models should have a CFI and TLI of at least 0.95.

For convergent validity, we investigated inter-correlations of the items of the CTS with the ACE [31]. Because of the ordinal nature of the data and non-normality, Kendall’s Tau was calculated.

To obtain normative data for the CTS, age- and gender-specific percentiles were generated for each CTS item, the total score, and the subscales. Percentiles were used because they are independent of the distribution of scale scores. Percentiles indicate the subject’s rank compared to other subjects of the same age group and gender, using a hypothetical group of 100 subjects. The sample size was sufficient to be divided into gender-specific age groups of ten years each for better clarity. Statistical analyses were conducted using SPSS, Version 21 and MPLUS, Version 7.3 [25]. Due to the large number of participants, the two subsamples differ significantly in the CTS items (emotional neglect *χ*^2^ = 57.3, physical neglect *χ*^2^ = 57.2, physical abuse *χ*^2^ = 57.8, emotional abuse *χ*^2^ = 34.5 and sexual abuse *χ*^2^ = 60.2) but only with very small effect sizes (emotional neglect Cramer’s V = 0.11, physical neglect Cramer’s V = 0.11 physical abuse Cramer’s V = 0.12, emotional abuse Cramer’s V = 0.08 and sexual abuse Cramer’s V = 0.11).

## Results

### Sample characteristics

Sample characteristics of the total sample and the two subsamples of 2013 and 2018 are presented in Table 1.

**Table 1:** Demographic characteristics of total sample and subsamples from the general population

	Total Sample (N = 5039)	Sample of 2013 (n = 2508)	Sample of 2018 (n = 2531)
Gender			
Female, N (%)	2735 (54.3)	1334 (53.2)	1401 (55.4)
Male, N (%)	2304 (45.7)	1174 (46.8)	1130 (44.6)
Age			
M	49.1	49.7	48.6
SD	18.2	18.3	18.0
Age-group			
14–20 years	288 (5.7)	141 (5.6)	147 (5.8)
21–30 years	712 (14.1)	335 (13.4)	337 (14.9)
31–40 years	709 (14.1)	339 (13.5)	370 (14.6)
41–50 years	871 (17.3)	456 (18.2)	415 (16.4
51–60 years	962 (19.1)	450 (17.9)	512 (20.2)
61–70 years	807 (16.0)	410 (16.3)	397 (15.7)
> 70 years	687 (13.7)	377 (15.0)	310 (12.2)
Living with a partner			
Yes	2666 (53.2)	1315 (52.4)	1351 (54.0)
No	2356 (46.8)	1193 (47.6)	1163 (46.0)
Education			
Did not graduate from school	123 (2.4)	67 (2.7)	56 (2.2)
Graduated school	4453 (88.6)	2221 (88.6)	2232 (88.6)
University degree	453 (9.0)	220 (8.8)	233 (9.2)
Employment status			
In training	403 (8.0)	192 (7.7)	211 (8.3)
Full-time employment	2063 (40.9)	996 (39.7)	1067 (42.2)
Part-time employment	678 (13.5)	310 (12.4)	368 (14.6)
Military or civilian service, maternal leave	45 (0.9)	19 (0.8)	26 (1.0)
Unemployment	267 (5.3)	142 (5.7)	125 (4.9)
Homemaker	183 (3.6)	104 (4.1)	79 (3.1)
Retired	1385 (27.5)	745 (29.7)	640 (25.3)
Unemployment			
Yes	2065 (42.7)	1021 (42.1)	1044 (47.6)
No	2776 (57.3)	1451 (57.9)	1325 (52.4)
Household income			
< 750€/month	185 (3.8)	114 (4.7)	71 (2.9)
750–1249 €/month	712 (14.6)	403 (16.6)	309 (12.2)
1250–1999€/month	1347 (27.6)	735 (30.2)	612 (24.2)
> 2000€/month	2635 (54.0)	1180 (48.5)	1455 (60.7)
Nationality %			
German	4834 (95.9)	2412 (96.2)	2422 (95.7)
Other	205 (4.1)	96 (3.8)	109 (4.3)

A total of 9453 valid addresses were identified (4360 in 2013 and 5093 in 2016). The main reasons for non-participation were that it was not possible to reach someone in the residence (after four attempts: 2013: 12.9%, 2018: 14.4%), that the person who answered the door refused to let anyone in the household participate in the study (2013: 13.6%; 2016: 16.5%), that it was not possible to contact the randomly selected household member (2013: 1.9%, 2018: after four attempts: 2.6%) and that the selected member refused to participate in the study (2013: 12.4%; 2018: 15.8%).

The final sample counted 5039 participants. Missing values were low. For example, only 14 respondents did not complete the CTS. Of the total sample, 54.3% were female. The mean age was 49.1 years (SD = 18.2). The sample characteristics closely match those of the German population in gender (54.3% female vs. 50.8%), employment status (unemployed: 5.3% vs. 5.7%), and educational level (Statistisches) [26]. However, compared to the general population, subjects of non-German nationality were underrepresented in our study sample (4.1% vs 11.1%).

### Item characteristics, internal consistency and factorial validity

The item characteristics are presented in Table 2.

**Table 2:** Item characteristics of the CTS in the general population

	1 Never true			2 Rarely true			3 Sometimes true			4 Often true			5 Very often true				
	N	%	Σ%	N	%	Σ%	N	%	Σ%	N	%	Σ%	N	%	Σ%	M	SD
Emotional neglect^a^																	
Total (N = 5019)	2055	40.9	40.9	2034	40.5	81.4	579	11.5	92.9	282	5.6	98.4	69	1.4	100	1.86	0.92
Male (N = 2295)	829	36.1	36.1	1007	43.9	80.0	293	12.8	92.8	140	6.1	98.9	26	1.1	100	1.92	0.91
Female (N = 2724)	1226	45.0	45.0	1027	37.7	82.7	286	10.5	93.2	142	5.2	98.4	43	1.6	100	1.81	0.93
Physical neglect^b^																	
Total (N = 5012)	2508	50.0	50.0	1244	24.8	74.8	676	13.5	88.3	220	4.4	92.7	364	7.2	100	1.94	1.21
Male (N = 2293)	1096	47.8	47.8	593	25.9	73.7	347	15.1	88.8	87	3.8	92.6	170	7.4	100	1.92	1.20
Female (N = 2719)	1412	51.9	51.9	651	23.9	75.8	329	12.1	87.9	133	4.9	92.8	194	7.1	100	1.91	1.21
Physical abuse^c^																	
Total (N = 5018)	3904	77.8	77.8	607	12.1	89.9	336	6.7	96.6	137	2.7	99.3	34	0.7	100	1.36	0.78
Male (N = 2292)	1727	75.3	75.3	309	13.5	88.8	173	7.5	96.3	69	3.0	99.3	14	0.6	100	1.40	0.80
Female (N = 2726)	2177	79.9	79.9	298	10.9	90.8	163	6.0	96.8	68	2.5	99.3	20	0.7	100	1.33	0.76
Emotional abuse^d^																	
Total (N = 5020)	3999	79.7	79.7	579	11.5	91.2	292	5.8	97	119	2.4	99.4	31	0.6	100	1.33	0.74
Male (N = 2294)	1828	79.7	79.7	280	12.2	91.9	128	5.6	97.5	46	2.0	99.5	12	0.5	100	1.31	0.71
Female (N = 2726)	2171	79.6	79.6	299	11.0	90.6	164	6.0	96.6	73	2.7	99.3	19	0.7	100	1.34	0.77
Sexual abuse^e^																	
Total (N = 5020)	4679	93.2	93.2	152	3.0	96.2	127	2.5	98.7	44	0.9	99.6	18	0.4	100	1.12	0.50
Male (N = 2293)	2199	95.9	95.9	42	1.8	97.7	33	1.4	99.1	14	0.6	99.7	5	0.2	100	1.07	0.40
Female (N = 2727)	2480	90.9	90.9	110	4.0	94.9	94	3.4	98.3	30	1.1	99.4	13	0.5	100	1.16	0.57

*M* mean, *SD* standard deviation, *N* number, *Σ*% cumulative percent

The items for the assessment of the respective type of maltreatment were:

When I was growing up…

^a^I felt loved (R)

^b^There was someone to take me to the doctor when I needed it (R)

^c^People in my family hit me so hard; it left me with bruises or marks

^d^I felt that somebody in my family hated me

^e^Somebody molested me

The inter-correlations between the items ranged between 0.17 for physical neglect and sexual abuse and 0.57 for emotional abuse and physical abuse, indicating small to strong effect sizes [6]. The inter-correlations are presented in Table 3.

**Table 3:** Spearman Rho Intercorrelation between the CTS items in the general population

	Emotional neglect	Physical neglect	Physical abuse	Emotional abuse	Sexual abuse
Emotional neglect	1.00	0.41^**^	0.43^**^	0.44^**^	0.24^**^
Physical neglect		1.00	0.21^**^	0.23^**^	0.17^**^
Physical abuse			1.00	0.57^**^	0.34^**^
Emotional abuse				1.00	0.38^**^
Sexual abuse					1.00

^*^p < 0.05, **p < 0.01, ***p < 0.001

Considering the brevity of the two subscales, the CTS total scale (α = 0.68) and the CTS abuse subscale (α = 0.73) showed acceptable internal consistencies. However, the neglect subscale showed poor internal consistency (α = 0.50). An additional table shows this in more detail (see Additional file 1).

**Fig. 1:**
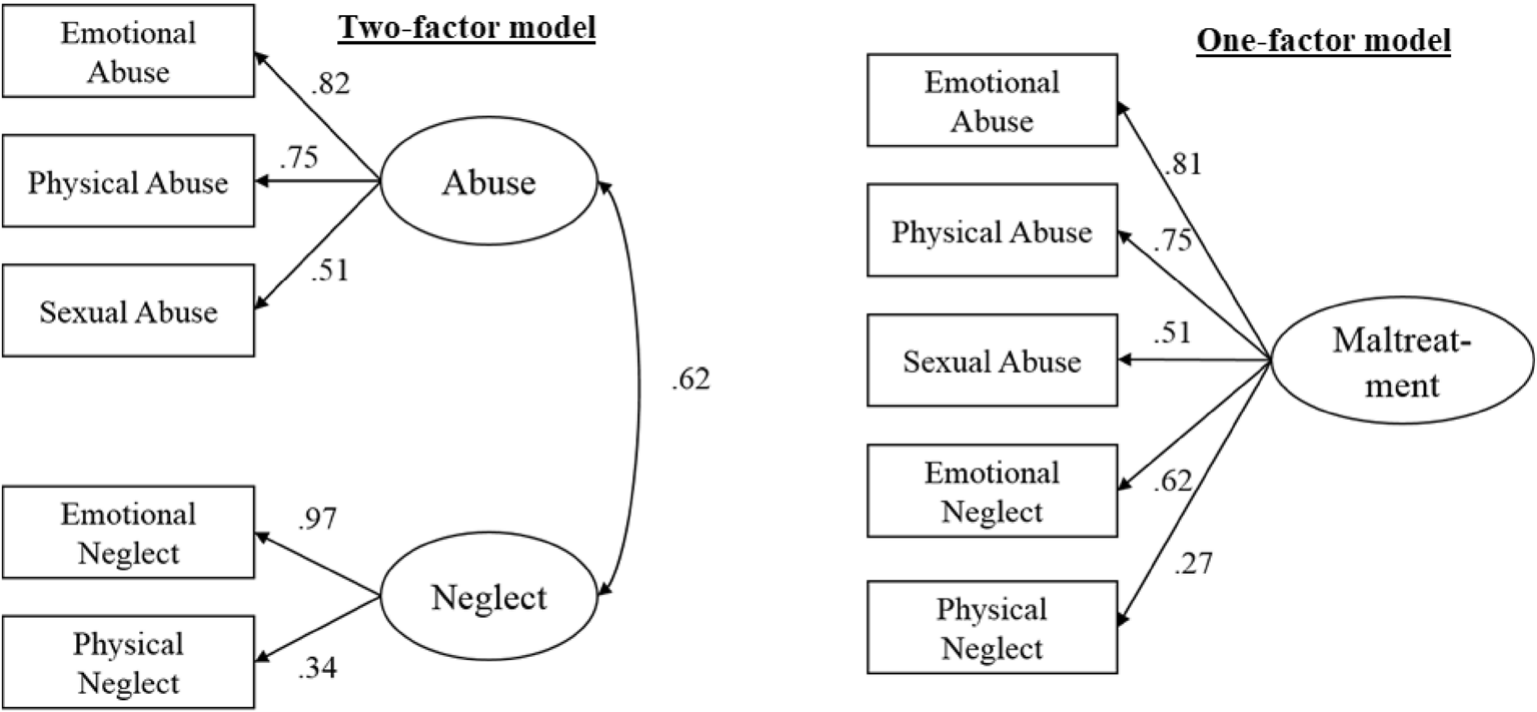
Results of the confirmatory factor analyses for a one- and two factor model

To evaluate the dimensional structure of the CTS, confirmatory factor analyses (CFA) was conducted. Therefore, a 1-factor model including all five CTS items was estimated and compared with a two-factor Model with loadings from the two neglect items onto the neglect factor and the three abuse items with factor loadings on an abuse factor. As shown in Fig. 1, factor loadings ranged from 0.34 to 0.82.

Compared with the one-factor model, the suggested two-factor model fits the data better, as indicated by robust fit indices CFI = 0.986, TLI = 0.994, RMSEA = 0.041, and the 90% confidence interval for RMSEA = 0.029–0.053 (model 1 in Table 4). A second step was to test if the model parameters would vary between men and women. Therefore, the total sample was split into males and females. A multigroup CFA was used to evaluate if factor loadings, residuals and model fit would differ between those four groups (Table 4). Chi-square values were used to examine structural invariance between gender groups. Results for the one-factor solution showed less favorable TLI and CFI indices (0.875 and 0.938, respectively). In line with the analyses of the total sample, the comparison for the gender subgroups also indicated a two-factor solution to fit the data best. Generally, the factor loading of the physical neglect item was relatively small.

**Table 4:** Multigroup confirmatory factor analysis (CFA) in four subsamples of females and males

CFA models	Fit indices for different CFA Models					Comparison between models		
	RMSEA	90% CI		TLI	CFI	df	ΔChi^2^	p
Two factor model	0.041	0.029	0.053	0.986	0.994	1	338.332^a^	< 0.001
Two factor model male	0.055	0.038	0.073	0.973	0.989	1	170.445^b^	< 0.001
Two factor model female	0.029	0.012	0.047	0.994	0.997	1	166.703^c^	< 0.001
One factor model	0.121	0.111	0.132	0.875	0.938			
One factor model male	0.130	0.115	0.146	0.849	0.925			
One factor model female	0.114	0.100	0.129	0.896	0.948			

^a^Difference comparison between one and two factor model total

^b^Difference comparison between one and two factor model male

^c^Difference comparison between one and two factor model female

### Convergent validity

To assess convergent validity, inter-correlations (Kendall’s Tau, *τ*) between the CTS items and the respective items of ACE-D were calculated. The correlation of the emotional neglect items was *τ* = 0.401 (*p* < 0.001), for the physical neglect items was *τ* = 0.161 (*p* < 0.001), for the physical abuse items was *τ* = 0.629 (*p* < 0.001), for the emotional abuse items was *τ* = 0.489 (*p* < 0.001), for the sexual abuse items was *τ* = 0.619 (*p* < 0.001) and for the total scale of the CTS and the sum score of the five ACE items was *τ* = 0.406 (*p* < 0.001).

### Normative data

Table 5 summarizes the normative data for the different age groups stratified by gender. Data for the CTS total score, the abuse subscale score and the neglect subscale score are presented. This data can be used to compare individual scores and scores from specific populations with normative data from the general population.

**Table 5:** Normative data from the general population for CTS

	Male							Female							Total
	14–20 n = 141	21–30 n = 331	31–40 n = 326	41–50 n = 337	51–60 n = 459	61–70 n = 362	> 70 n = 330	14–20 n = 146	21–30 n = 378	31–40 n = 383	41–50 n = 496	51–60 n = 500	61–70 n = 441	> 70 n = 379	n = 4992
	Σ %	Σ %	Σ %	Σ %	Σ %	Σ %	Σ %	Σ %	Σ %	Σ %	Σ %	Σ %	Σ %	Σ %	Σ %
Neglect subscale															
2	51.8	48.2	45.2	34.7	38.4	36.9	36.2	55.2	51.9	48.8	46.6	44.5	42.2	36.1	43.2
3	75.9	70.0	69.5	62.6	62.0	59.4	57.9	78.6	73.8	68.3	70.0	66.1	67.8	58.6	66.3
4	85.8	80.9	81.8	76.2	77.9	76.7	74.3	88.3	84.4	79.4	80.0	78.4	80.5	72.6	79.1
5	87.9	87.3	88.0	83.7	86.0	84.2	83.2	91.7	87.8	86.3	85.2	86.0	87.1	83.9	86.0
6	97.2	96.1	96.3	93.5	96.3	97.2	98.0	98.6	95.8	97.1	97.6	96.0	97.1	97.1	96.6
7	99.3	99.1	98.5	97.8	99.1	98.3	99.3	99.3	99.7	99.5	98.8	98.4	98.6	98.9	98.9
8	99.9	99.7	99.7	99.7	99.6	99.2	99.7	99.9	99.9	99.9	99.9	99.6	99.5	99.5	99.7
9		99.9	99.9	99.9	99.8	99.9	99.9					99.9	99.8	99.9	99.9
10					99.9							99.9	99.9		
Abuse subscale															
3	72.7	73.0	72	64.2	68.8	68.6	63.7	69.2	70.1	70.3	71.4	71.3	70.9	69.0	69.6
4	85.6	85.5	83.1	78.0	80.4	82.8	79.2	86.3	79.9	80.8	80.0	80.5	82.8	80.7	81.3
5	89.9	91.8	90.8	85.6	85.4	90.8	89.1	91.8	87.6	86.9	86.7	86.5	88.7	88.1	88.1
6	93.5	93.3	92.6	89.2	91.9	93.6	93.7	93.8	91.8	91.1	91.5	90.0	91.9	91.5	91.8
7	97.1	96.1	95.1	93.2	94.8	96.7	97.4	95.2	95.5	94.2	94.4	92.8	95.5	94.2	94.9
8	97.8	96.7	97.5	96.5	97.8	97.8	98.7	95.9	96.6	95.5	95.6	95.0	97.1	96.3	96.7
9	98.6	98.8	98.8	98.1	98.9	98.6	99.0	97.9	97.4	98.2	97.8	97.0	98.2	98.7	98.2
10	99.9	99.1	99.4	98.9	99.1	98.9	99.3	99.9	98.4	99.0	99.4	98.2	98.9	99.2	99.0
11	99.9	99.7	99.7	99.2	99.8	99.7	99.7		99.5	99.7	99.6	98.8	99.5	99.5	99.5
12	99.9	99.9	99.9	99.7	99.9	99.9	99.9		99.9	99.9	99.9	99.2	99.8	99.7	99.7
13												99.6	99.9	99.9	99.9
14												99.9			
15															
Total score															
5	23.0	25.8	31.1	20.7	23.1	20.9	19.8	33.1	32.0	33.3	28.5	30.5	28.9	19.7	26.5
6	54.0	51.8	46.8	38.7	38.6	37.9	36.3	57.2	53.7	49.2	49.2	47.0	46.2	37.5	45.2
7	74.1	67.3	66.8	58.3	61.1	55.4	51.8	73.8	71.2	62.4	64.2	61.6	64.5	54.5	62.4
8	77.7	75.8	75.1	69.2	70.3	66.0	64.4	82.8	75.7	70.6	72.5	70.9	74.0	65.7	71.5
9	83.5	83.0	83.4	77.9	78.8	76.6	75.6	90.3	83.6	79.6	79.6	78.9	80.6	77.1	80.0
10	90.6	91.2	88.0	83.7	86.0	86.4	84.2	91.7	87.8	85.7	85.4	84.7	87.0	85.6	86.5
11	92.8	93.3	90.8	87.2	88.6	90.3	90.4	95.2	90.5	89.7	88.3	89.4	89.3	89.1	89.7
12	95.7	93.9	92.9	89.6	91.7	92.8	93.1	96.6	92.1	91.8	90.9	91.8	92.5	91.5	92.2
13	96.4	95.8	93.8	93.2	94.5	95.5	96.7	97.2	94.2	93.9	93.7	94.0	94.1	95.2	94.6
14	97.1	96.4	96.3	94.8	96.5	97.8	98.0	98.6	95.5	96.3	95.3	95.2	95.9	96.8	96.2
15	99.3	98.2	96.9	96.7	98.3	98.3	99.0	99.9	96.6	97.9	97.0	96.6	97.3	99.2	97.7
16	99.9	98.8	99.1	98.6	99.1	98.6	99.7		98.1	98.9	98.0	98.4	98.2	99.5	98.7
17		99.1	99.9	99.5	99.8	98.9	99.9		98.9	99.5	99.0	98.6	98.9	99.7	99.2
18		99.4		99.9	99.9	99.2			99.5	99.7	99.9	99.0	99.3	99.9	99.5
19		99.9				99.4			99.7	99.9		99.4	99.9		99.7
20						99.7			99.9			99.9	99.9		99.9
21						99.9									
22															
23															
24															
25															

*n* number of participants, *Σ*% cumulative percentage of endorsement

## Discussion

Generally, based on more than 5000 participants from the German general population, our results demonstrate that the CTS, an ultrashort version of the CTQ, is a valid screening instrument for retrospective assessment of child maltreatment in the general population. The results also indicate that the physical neglect item has inadequate psychometric properties. Therefore, the use of this particular item is questionable. While data on validation [13] and clinical cut-offs [12] were available, this is the first study to provide norm data and, more importantly, to examine the psychometric properties of the CTS in isolation from the CTQ. Previous studies have examined the psychometric properties of the CTS only in conjunction with the CTQ. It should be considered that these psychometric properties may change when presented separately and not in conjunction with another twenty items that also measure child maltreatment.

Due to the brevity of the CTS and the general interrelatedness of different types of maltreatment [16, 32], it was not possible to test whether each item represented a separate scale. However, the CFA revealed evidence of a two-factor structure of the questionnaire with excellent indicators of model fit. This factor structure is consistent with the Centers for Disease Control's (CDC) classification of child maltreatment, which has established consistent definitions of child maltreatment across professions [22]. In this classification, child maltreatment is split into acts of commission, including emotional maltreatment, physical maltreatment, and sexual abuse, and acts of omission, including failure to assist and failure to supervise. The CFA results are largely consistent with this classification, underscoring the construct validity of the instrument. The correlation between the two factors (see Fig. 1) is also evidence of the general correlation between the different types of maltreatment. Therefore, it can be assumed that the total score and the abuse score (emotional, physical and sexual abuse) and the neglect score (emotional and physical neglect) can be calculated and used in analyses. It should be noted, however, that the neglect scale has insufficient psychometric properties (Cronbach's alpha 0.5) and should therefore be used with caution. In contrast, the abuse subscale showed acceptable internal consistency (Cronbach’s alpha of 0.73) [28]. It is worth noting that such ultra-short measures do not have psychometric properties comparable to longer measures such as the CTQ. However, retrospective assessment of physical neglect appears to be problematic in general, as other studies have also criticized the reliability of the physical neglect subscale of the CTQ [11, 21].

The convergent validity of the CTS is generally supported by high intercorrelations of the CTS items with the corresponding items from the ACE. The sum score also showed moderate correlation with the corresponding score extracted from the ACE. Therefore, the convergent validity of the CTS can be assumed. Once again, the physical neglect item showed only a low correlation, underscoring that this item may not be suitable for reliably and validly assessing physical neglect, which is possibly due to cultural and socioeconomic influences. A recent prevalence study of child maltreatment in the German general population based on the CTQ [32] reports prevalence rates for at least moderate severity. For physical neglect, the authors report a rate of 22.4% for emotional neglect, 13.3% for physical neglect, 6.6% for physical abuse, 6.5% for emotional abuse and 7.6% for sexual abuse. Setting a cut-off of at least 3 in the present study results to comparable rates, with 18.6% for emotional neglect, 25.1% for physical neglect, 10.1% for physical abuse, 8.8% for emotional abuse, with the exception of sexual abuse with a rate of 3.8%. These results suggest that the sexual abuse item may be less sensitive to sexual abuse assessment than when sexual abuse is assessed with more items. Overall, research suggests that measures with more items result in higher prevalence rates of child sexual abuse [27] and therefore may also more accurately capture this type of maltreatment. When assessing child maltreatment, extensive measures should be used whenever possible. However, circumstances such as economic considerations may necessitate the use of an ultrashort measure, such that the use of this measure would be justified. This may be particularly true when child maltreatment is not the primary outcome variable but is used as a control variable.

By reporting normative data, we provide the opportunity to contextualize individual outcomes and specific clinical samples. Given that age- and gender-specific comparative data were generated based on subgroups of 141–500 participants, sample sizes were sufficient to provide normative data for these subgroups. In general, scores of > 2 on the abuse items should be considered a warning signal. However, as mentioned above, the sexual abuse scale may be less sensitive and therefore may result in lower prevalence rates. For this item in particular, values of more than one should be regarded as a warning signal. Due to the rather insufficient psychometric properties, the item on physical neglect should only be used with great caution.

Two of the strengths of this study are the large sample size and the representativeness of the study sample. However, this study also has some weaknesses. The data for this study were combined from two samples from 2013 and 2018 using identical methods and instruments. However, we found that the CTS items yielded slightly lower prevalence rates in 2018 than in 2013. It would appear that these fluctuations are more likely to be due to normal statistical variation. It is less likely that this can be interpreted as an actual change in prevalence rates or that it is due to a change in social norms. Moreover, the order of presentation might also have contributed to these differences. Therefore, combining the two samples could reconcile the differences in prevalence rates and lead to a more accurate estimate of CTS norms. Another potential limitation of the study is the response rate of 78.9%. In general, response rates are lower in general population studies than in clinical trials. Although the random route approach is a very established method, particularly due to its economic and practical qualities, it also has its limitations. Bauer [2, 3] showed in his calculations that it violates the assumption of equal probability and that this leads to distorted expected values for several variables. The strongest errors were found in variables related to the spatial location of households. This means that the method provides good indications of the occurrence of variables in the population, but there may be bias if there is a strong local occurrence. Therefore, the study systematically excludes potential high-risk populations, such as, individuals with inadequate German language skills and individuals currently living in institutions. Another limitation is the Underrepresentation of other nationalities and refugees.In addition, validity tests for an instrument must demonstrate both convergent and discriminant validity. This study for the general population did not include broader measures of child maltreatment or multi-informant measures, which are thought to result in the highest rates. Nevertheless, convergent validity with the German version of the ACE [31] was demonstrated, except for the item assessing physical neglect. Another possible limitation could be the items for emotional neglect and emotional abuse, which reflect the subject's emotional feelings rather than representing objective behavior. The CTS is not sufficient for a comprehensive assessment of child maltreatment. However, it may provide useful information in the context of research that includes child maltreatment as a control variable. Overall, longer instruments such as the CTQ should be preferred for retrospective assessment of child maltreatment. However, in certain circumstances, a shorter instrument may be necessary. The use of the CTS is recommended as an effective instrument in settings where resources are strictly limited. Future studies should focus primarily on a more reliable and valid assessment of physical neglect and a more sensitive assessment of sexual abuse, as prevalence is highly dependent on the assessment method, the definition used or the form of sexual abuse (e.g. hands-on, hands-off acts) [1, 9, 27].

## Supplementary Information

**Additional file 1: **Item characteristics of the CTS scales in the general population.

## Data Availability

The datasets generated and/or analyzed during the current study are not publicly available but are available from the corresponding author on reasonable request.

## Abbreviations

ACE: Adverse childhood experience
CDC: Centers for Disease Control and Prevention
CFA: Confirmatory factor analysis
CFI: Comparative Fit Index
CTS: Childhood Trauma Screener
CTQ: Childhood Trauma Questionnaire
RMSEA: Root mean square error of approximation
SPSS: Statistical Package for the Social Sciences
TLI: Tucker–Lewis Index
