# Supplementary material for: Validation and standardization of the Childhood Trauma Screener (CTS) in the general population
Source: Child Adolesc Psychiatry Ment Health. 2022 Sep 1;16:73. doi: 10.1186/s13034-022-00506-6 (PMC9438230; doi:10.1186/s13034-022-00506-6)
Supplement: Supplementary file 1 — Additional file 1: Item characteristics of the CTS scales in the general population. [file 13034_2022_506_MOESM1_ESM.docx]

Additional file 1.

Item characteristics of the CTS scales in the general population.

|  | | Total scale | | | Abuse subscale | | | Neglect subscale | | |
| --- | --- | --- | --- | --- | --- | --- | --- | --- | --- | --- |
|  |  | Total  (N=4992) | Male  (N=2283) | Female  (N=2709) | Total  (N=4992) | Male (N=2283) | Female  (N=2709) | Total  (N=4992) | Male (N=2283) | Female  (N=2709) |
|  | M | 7.6 | 7.7 | 7.6 | 3.8 | 3.8 | 3.8 | 3.3 | 3.4 | 3.2 |
|  | SD | 2.86 | 2.76 | 2.95 | 1.66 | 1.58 | 1.73 | 1.55 | 1.56 | 1.53 |
| 1 | N |  |  |  |  |  |  |  |  |  |
|  | % |  |  |  |  |  |  |  |  |  |
|  | Σ% |  |  |  |  |  |  |  |  |  |
| 2 | N |  |  |  |  |  |  | 2163 | 926 | 1237 |
|  | % |  |  |  |  |  |  | 43.2 | 40.5 | 45.5 |
|  | Σ% |  |  |  |  |  |  | 43.2 | 40.5 | 45.5 |
| 3 | N |  |  |  | 3488 | 1569 | 1919 | 1156 | 543 | 613 |
|  | % |  |  |  | 69.6 | 68.6 | 70.5 | 23.1 | 23.7 | 22.6 |
|  | Σ% |  |  |  | 69.6 | 68.6 | 70.5 | 66.3 | 64.2 | 68.1 |
| 4 | N |  |  |  | 586 | 298 | 288 | 641 | 326 | 315 |
|  | % |  |  |  | 11.7 | 13.0 | 10.6 | 12.8 | 14.2 | 11.6 |
|  | Σ% |  |  |  | 81.3 | 81.6 | 81.1 | 79.1 | 78.4 | 79.7 |
| 5 | N | 1325 | 535 | 790 | 339 | 162 | 177 | 343 | 163 | 180 |
|  | % | 26.5 | 23.4 | 29.2 | 6.8 | 7.1 | 6.5 | 6.9 | 7.1 | 6.6 |
|  | Σ% | 26.5 | 23.4 | 29.2 | 88.1 | 88.7 | 87.6 | 86.0 | 85.5 | 86.3 |
| 6 | N | 932 | 428 | 504 | 186 | 83 | 103 | 531 | 245 | 286 |
|  | % | 18.7 | 18.7 | 18.6 | 3.7 | 3.6 | 3.8 | 10.6 | 10.7 | 10.5 |
|  | Σ% | 45.2 | 42.2 | 47.8 | 91.8 | 92.3 | 91.4 | 96.6 | 96.2 | 96.9 |
| 7 | N | 860 | 429 | 431 | 155 | 73 | 82 | 114 | 57 | 57 |
|  | % | 17.2 | 18.8 | 15.8 | 3.1 | 3.2 | 3.0 | 2.3 | 2.5 | 2.1 |
|  | Σ% | 62.4 | 61.0 | 63.6 | 94.9 | 95.5 | 94.4 | 98.9 | 98.7 | 99.0 |
| 8 | N | 450 | 218 | 232 | 87 | 45 | 42 | 40 | 19 | 21 |
|  | % | 9.0 | 9.5 | 8.6 | 1.7 | 2.0 | 1.5 | 0.8 | 0.8 | 0.8 |
|  | Σ% | 71.5 | 70.5 | 72.2 | 96.7 | 97.5 | 96.0 | 99.7 | 99.6 | 99.7 |
| 9 | N | 425 | 20.3 | 222 | 78 | 27 | 51 | 14 | 9 | 5 |
|  | % | 8.5 | 8.9 | 8.2 | 1.6 | 1.2 | 1.9 | 0.3 | 0.3 | 0.2 |
|  | Σ% | 80.0 | 79.4 | 80.4 | 98.2 | 98.7 | 97.8 | 99.9 | 99.9 | 99.9 |
| 10 | N | 326 | 167 | 159 | 40 | 11 | 29 | 3 | 1 | 2 |
|  | % | 6.5 | 7.3 | 5.9 | 0.8 | 0.5 | 1.1 | 01. | 0.1 | 0.1 |
|  | Σ% | 86.5 | 86.7 | 86.3 | 99.0 | 99.2 | 98.9 | 100 | 100 | 100 |
| 11 | N | 162 | 77 | 85 | 26 | 11 | 15 |  |  |  |
|  | % | 3.2 | 3.4 | 3.1 | 0.5 | 0.5 | 0.6 |  |  |  |
|  | Σ% | 89.7 | 90.1 | 89.4 | 99.5 | 99.7 | 99.4 |  |  |  |
| 12 | N | 121 | 54 | 67 | 9 | 3 | 6 |  |  |  |
|  | % | 2.4 | 2.4 | 2.5 | 0.2 | 0.1 | 0.2 |  |  |  |
|  | Σ% | 94.6 | 92.5 | 91.9 | 99.7 | 99.8 | 99.7 |  |  |  |
| 13 | N | 121 | 57 | 64 | 7 | 2 | 5 |  |  |  |
|  | % | 2.4 | 2.5 | 2.4 | 0.1 | 0.1 | 0.2 |  |  |  |
|  | Σ% | 94.6 | 95.0 | 94.3 | 99.9 | 99.9 | 99.9 |  |  |  |
| 14 | N | 81 | 38 | 43 | 3 | 2 | 1 |  |  |  |
|  | % | 1.6 | 1.7 | 1.6 | 0.1 | 0.0 | 0.0 |  |  |  |
|  | Σ% | 96.2 | 96.6 | 95.9 | 99.9 | 99.9 | 99.9 |  |  |  |
| 15 | N | 73 | 31 | 42 | 4 | 1 | 3 |  |  |  |
|  | % | 1.5 | 1.4 | 1.6 | 0.1 | 0.1 | 0.1 |  |  |  |
|  | Σ% | 97.7 | 98.0 | 97.4 | 100 | 100 | 100 |  |  |  |
| 16 | N | 49 | 21 | 28 |  |  |  |  |  |  |
|  | % | 1.0 | 0.9 | 1.0 |  |  |  |  |  |  |
|  | Σ% | 98.7 | 98.9 | 98.4 |  |  |  |  |  |  |
| 17 | N | 27 | 13 | 14 |  |  |  |  |  |  |
|  | % | 0.5 | 0.6 | 0.5 |  |  |  |  |  |  |
|  | Σ% | 99.2 | 99.5 | 99.0 |  |  |  |  |  |  |
| 18 | N | 17 | 5 | 12 |  |  |  |  |  |  |
|  | % | 0.3 | 0.2 | 0.4 |  |  |  |  |  |  |
|  | Σ% | 99.5 | 99.7 | 99.4 |  |  |  |  |  |  |
| 19 | N | 10 | 4 | 6 |  |  |  |  |  |  |
|  | % | 0.2 | 0.2 | 0.2 |  |  |  |  |  |  |
|  | Σ% | 99.7 | 99.9 | 99.6 |  |  |  |  |  |  |
| 20 | N | 6 | 1 | 5 |  |  |  |  |  |  |
|  | % | 0.1 | 0 | 0.2 |  |  |  |  |  |  |
|  | Σ% | 99.9 | 99.9 | 99.8 |  |  |  |  |  |  |
| 21 | N | 2 | 1 | 1 |  |  |  |  |  |  |
|  | % | 0.0 | 0.0 | 0.1 |  |  |  |  |  |  |
|  | Σ% | 99.9 | 99.9 | 99.9 |  |  |  |  |  |  |
| 22 | N | 1 | 1 | 4 |  |  |  |  |  |  |
|  | % | 0.0 | 0.0 | 0.1 |  |  |  |  |  |  |
|  | Σ% | 99.9 | 100 | 100 |  |  |  |  |  |  |
| M=mean, SD=standard deviation, N=number, Σ%=cumulative percent | | | | | | | | | | |
